# Comparing validity of Edinburgh scale and SRQ20 in screening for post-partum depression

**DOI:** 10.1186/1745-0179-3-18

**Published:** 2007-09-28

**Authors:** Iná S Santos, Alicia Matijasevich, Beatriz F Tavares, Andrey C da Cruz Lima, Rafael E Riegel, Bruna C Lopes

**Affiliations:** 1 Department of Social Medicine, Faculty of Medicine, Federal University of Pelotas, Pelotas, Brazil; 2 Department of Mental Health, Faculty of Medicine, Federal University of Pelotas, Pelotas, Brazil

## Abstract

The Edinburgh Postnatal Depression Scale (EPDS) is the instrument most used worldwide for screening of Post-Partum Depression (PPD). The SRQ20 questionnaire has been largely used for screening of minor psychiatric disorders. This study aimed to compare the accuracy of the two instruments in screening for PPD. At the third-month follow-up home visit to infants of the 2004 Pelotas Birth Cohort, Southern Brazil, a sub-sample of 378 mothers was selected. Among other questions, EPDS and SRQ20 were applied by trained fieldworkers. Up to 15 days later, a mental health professional re-interviewed the mother (the gold standard interview). Sensitivity and specificity of each cutoff point were calculated for EPDS and SRQ20 and the results were plotted at a ROC curve. The areas under both curves were compared. Highest sensitivity and specificity cutoff were observed for EPDS ≥ 10 (sensitivity 82.7%, 95%CI 74.0 – 89.4; specificity 65.3%, 95%CI 59.4 – 71.0) and for SRQ20 ≥ 6 (sensitivity 70.5%, 95%CI 60.8 – 79.0%; specificity 75.5%, 95%CI 70.0 – 80.5%). Shape of ROC curves and areas under both curves were virtually identical (respectively, 0.8401 ± 0.02 for EPDS and 0.8402 ± 0.02 for SRQ20; p = 0.9). In conclusion SRQ20 showed to be as valid as EPDS as a screening tool for PPD at third month after delivery.

## Background

Postpartum depression (PPD) can lead to maternal reduced interaction and irritability misdirected at the child [1]. PPD can impair adequate infant care and increase the risk for infant cognitive and emotional delay [2]. Some authors have recognized PPD as a public health problem and recommended the systematic screening of the condition, in both antenatal and postnatal periods [3,4]. In clinical settings, identification of PPD can be improved by increasing awareness and skills of health professionals in recognizing maternal depressive symptoms or by screening for PPD through the use of specific questionnaires [5].

The Edinburgh Postnatal Depression Scale (EPDS) [6] and the Self-Reporting Questionnaire 20 items (SRQ20) [7,8] are largely used in mental health research. The EPDS is the most widely accepted screening scale used worldwide in the perinatal period [9]. The SRQ20 was developed by the World Health Organization to screen for common mental disorders (depression and anxiety) in primary health care. Both questionnaires are not substitute for, or equivalent to, a clinical diagnosis. They indicate probable cases of mental disorder by identifying individuals who need further evaluation. Both tests are not of exclusive use among specific population groups. As sated by the authors, EPDS may be used for screening and diagnosis of depression at community level, indistinctly among men and women [6]. The SRQ20 questionnaire may be applied to identify in risk individuals, independently of sex and childbearing status.

This study was planned to compare the performance of SRQ20 and EPDS in screening for PPD in a sample of mothers from the 2004 Pelotas Birth Cohort, three months after delivery, having as gold standard an interview conducted by mental health professionals.

At age three months, all the infants from the cohort [10], a population-based study which included all children born in the city's five hospitals, were visited at home. At this occasion their mothers were interviewed and, among other questions the EPDS (Table 1) and the SRQ20 questionnaires were administered. The EPDS performance as a screening and diagnostic tool for post-partum depression was tested and the results are available in another publication [11].

**Table 1:** Edinburgh Test (Portuguese version)

Marque a resposta que melhor reflete como você tem se sentido nos últimos 7 dias:
1. Eu tenho sido capaz de rir e achar graça das coisas.
() Como eu sempre fiz.
() Não tanto quanto antes.
() Sem dúvida, menos que antes.
() De jeito nenhum.
2. Eu tenho pensado no futuro com alegria.
() Sim, como de costume.
() Um pouco menos que de costume.
() Muito menos que de costume.
() Praticamente não.
3. Eu tenho me culpado sem razão quando as coisas dão errado.
() Não, de jeito nenhum.
() Raramente.
() Sim, às vezes.
() Sim, muito freqüentemente.
4. Eu tenho ficado ansiosa ou preocupada sem uma boa razão.
() Sim, muito seguido.
() Sim, às vezes.
() De vez em quando.
() Não, de jeito nenhum.
5. Eu tenho me sentido assustada ou em pânico sem um bom motivo.
() Sim, muito seguido.
() Sim, às vezes.
() Raramente.
() Não, de jeito nenhum.
6. Eu tenho me sentido sobrecarregada pelas tarefas e acontecimentos do meu dia-a-dia.
() Sim. Na maioria das vezes eu não consigo lidar bem com eles.
() Sim. Algumas vezes não consigo lidar bem como antes.
() Não. Na maioria das vezes consigo lidar bem com eles.
() Não. Eu consigo lidar com eles tão bem quanto antes.
7. Eu tenho me sentido tão infeliz que eu tenho tido dificuldade de dormir.
() Sim, na maioria das vezes.
() Sim, algumas vezes.
() Raramente.
() Não, nenhuma vez.
8. Eu tenho me sentido triste ou muito mal.
() Sim, na maioria das vezes.
() Sim, muitas vezes.
() Raramente.
() Não, de jeito nenhum.
9. Eu tenho me sentido tão triste que tenho chorado.
() Sim, a maior parte do tempo.
() Sim, muitas vezes.
() Só de vez em quando.
() Não, nunca.
10. Eu tenho pensado em fazer alguma coisa contra mim mesma.
() Sim, muitas vezes.
() Às vezes.
() Raramente.
() Nunca.

For the purpose of the present study, mothers whose babies reached age three months between 1 January and 31 March 2005 (born, therefore, between 1 October and 31 December 2004) were included. The majority of the interviews were strictly paid in the time frame between one week before and one week after the day in which the infant completed his/her third month of life (between weeks 12 and 14 after birth, in average at week 13.1 (SD = 0.6). Only women who gave birth to singletons were eligible.

Questions of the two instruments were posed to mothers by a trained interviewer, as a single block and in the same order as in the original instrument. The decision to pose the questions to mothers verbally instead of self-administered was due to the high proportion of mothers with little schooling. The administration of SRQ20 and EPDS as an interview is accepted by the instrument's authors and has already been used previously [7,8].

To the present analysis, all mothers scoring 9 points or more in the EPDS were selected. Then, a systematic sample of mothers scoring < 9 was obtained by recruiting every fourth mother in this condition. All the selected mothers underwent a diagnostic interview (gold standard).

For the diagnostic interview (gold standard), mothers were re-visited at home by a mental health professional (psychiatrist, psychologist, or psychiatry resident), previously trained for the administration of a semi-structured interview, which was based on ICD-10 diagnostic criteria [12] to explored the presence of depressed mood most of the days of the last two consecutive weeks. It was a standardized semi-structured questionnaire containing major obligatory symptoms, accessory symptoms, as well as criteria for differential diagnosis to be fulfilled during the interview. For standardization purposes, interviewers were trained before data collection. Training included the reading and discussion of the instrument and role-playing sections headed by a Senior psychiatrist, who also reviewed and codified all records from interview with the mothers. The diagnostic interview was aimed at detecting the current or recent (last 15 days) depressive episodes. According to the result of this interview, mothers were classified as 'normal' or 'positive,' the latter including those with mild, moderate or severe episodes of depression. Mental health professionals were blinded as to the score obtained by mothers using EPDS or SRQ20. Average time interval between EPDS/SRQ20 application and the gold standard interview was 18.3 (SD = 9.6) days (median = 16 days; range = 5 – 60 days).

For each EPDS and SRQ20 cutoff point, sensitivity (proportion of depressed mothers according to ICD-10 criteria that were correctly identified) and specificity (proportion of non-depressed mothers correctly identified as such) were calculated. The cutoff point showing simultaneously the highest sensitivity and specificity was determined using a Receiver Operator Characteristic (ROC) curve for both, the EPDS and the SRQ20. Plotted in the same graphic, the ROC curves were used to compare accuracy of SRQ20 and EPDS as estimated through the size of the area under each curve. Stata 9.1 software was used for all analyses.

The research protocol was approved by the Research Ethics Committee of the University of Pelotas Medical School and written maternal consent was obtained before the interviews.

A total of 965 singleinfants were born between 1 October and 31 December 2004. There were 79 losses and refusals (8.2%). Of the 886 mothers for whom the SRQ20 and EPDS were administered, 219 presented EPDS ≥ 9 and 667 EPDS < 9. As a result 378 responded also to the diagnostic interview (219 with EPDS ≥ 9 and 159 with EPDS < 9). According to the gold standard, 105 mothers presented mild, moderate, or severe episodes of depression, and the remaining 273 were classified as normal.

The wide majority of the mothers (83.6%) came from families with monthly family income of up to three minimum wages. About 67% were aged 20–34 years, and over one-fifth (22.2%) were adolescents. Only two mothers had never attended school, whereas 15% had between one and four, and about 40%, nine or more years of schooling. The majority of women were white (70.9%), and 81.2% lived with a husband or partner. A little more than one-third of mothers (38.4%) worked outside home during pregnancy. The majority of pregnancies were unplanned (67.2%). The prevalence of babies with low birth weight (< 2,500 grams) and preterm births (< 37 gestational weeks) (10,8% and 16.4%, respectively), as well as the frequency of all maternal characteristics examined in the sample, with the exception of smoking during pregnancy, were statistically similar to those of the 2004 cohort as a whole (n = 4,287 alive and singleton births). The prevalence of maternal smoking was higher in the sample (33.6% versus 25.1%; p < 0.001).

**Figure 1:**
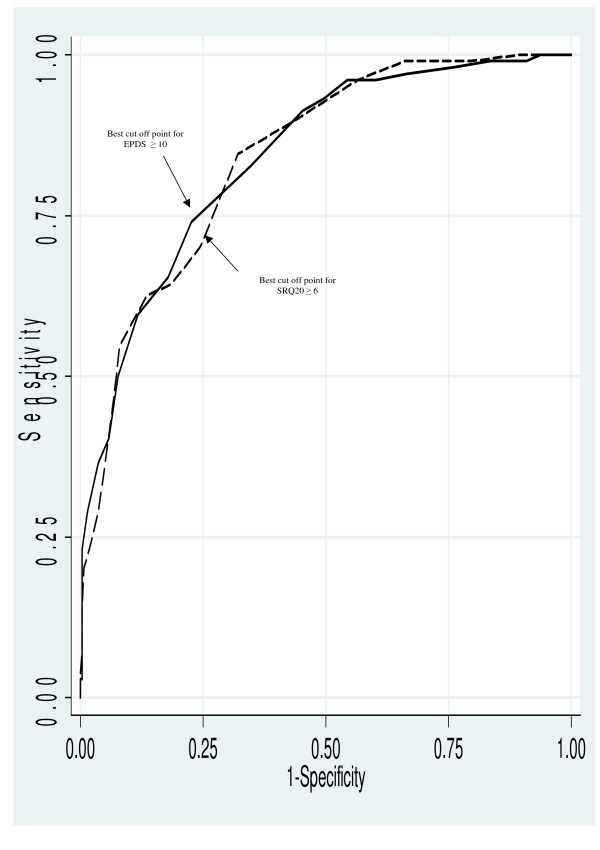
ROC curves for the SRQ20 and the EPDS for Post Partum Depression. Solid line: EPDS. Broken line: SRQ20

Correlation coefficient between EPDS and SRQ20 as regard to true positive results was 0.7251. The two tests fully agreed for 295 mothers. Among 83 mothers the observed disagreement was for 44 who were positive to the EPDS and negative to the SRQ20, and the contrary for the remaining 39 mothers. Kappa statistics of the agreement between the two tests was 56% (95%CI 48–64%). As shown in Table 2 and Figure 1, for EPDS the cutoff point ≥ 10 was the best for screening PPD among that population with sensitivity 82.7% (74.0% – 89.4%) and specificity 65.3% (59.4% – 71.0%). For SRQ20 the correspondent best cutoff was ≥ 6 with sensitivity 70.5% (95%CI 60.8 – 79.0%) and specificity 75.5% (95%CI 70.0 – 80.5%). At those cutoffs, accuracy of both instruments was similar, respectively, 70.1% for EPDS and 74.1% for SRQ20. Figure 1 also shows that shape of ROC curves and areas under both curves were virtually identical, respectively, 0.8401 ± 0.02 for EPDS and 0.8402 ± 0.02 for SRQ20 (p = 0.9).

**Table 2:** Sensitivity, specificity of a Brazilian version of EPDS and of SRQ20.

EPDS			SRQ20		
Cutoff points	Sensitivity (95%CI)	Specificity (95%CI)	Cutoff points	Sensitivity (95%CI)	Specificity (95%CI)

≥3	99.0 (94.8 – 100)	16.4 (12.2 – 21.4)	≥1	100 (96.5 – 100)	10.9 (7.5 – 15.3)
≥4	98.1 (93.3 – 99.8)	23.7 (18.8 – 29.2)	≥2	99.0 (94.8 – 100)	20.4 (15.8 – 25.7)
≥5	97.1 (91.9 – 99.4)	33.6 (28.0 – 39.5)	≥3	99.0 (94.8 – 100)	33.9 (28.4 – 39.9)
≥6	96.2 (90.5 – 99.0)	39.8 (33.9 – 45.8)	≥4	96.2 (90.5 – 99.0)	43.4 (37.5 – 49.5)
≥7	96.2 (90.5 – 99.0)	45.6 (39.6 – 51.7)	≥5	90.5 (83.2 – 95.3)	55.1 (49.0 – 61.1)
≥8	93.3 (86.7 – 97.3)	50.4 (44.3 – 56.4)	≥6	84.8 (76.4 – 91.0)	67.9 (62.0 – 73.4)
≥9	91.3 (84.2 – 96.0)	54.7 (48.6 – 60.7)	≥7	70.5 (60.8 – 79.0)	75.5 (70.0 – 80.5)
≥10	82.7 (74.0 – 89.4)	65.3 (59.4 – 71.0)	≥8	64.8 (54.8 – 73.8)	81.4 (76.3 – 85.8)
≥11	74.0 (64.5 – 82.1)	77.4 (72.0 – 82.2)	≥9	62.9 (52.9 – 72.1)	86.5 (81.9 – 90.3)
≥12	65.4 (55.4 – 74.4)	82.1 (77.1 – 86.5)	≥10	54.3 (44.3 – 60.0)	92.0 (88.1 – 94.9)
≥13	59.6 (49.5 – 69.1)	88.3 (83.9 – 91.9)	≥11	44.8 (35.0 – 54.8)	93.4 (89.8 – 96.1)
≥14	50.0 (40.0 – 60.0)	92.3 (88.5 – 95.2)	≥12	28.6 (20.2 – 38.2)	96.4 (93.4 – 98.2)
≥15	40.4 (30.9 – 50.5)	94.2 (90.7 – 96.6)	≥13	22.9 (15.2 – 32.1)	98.2 (95.8 – 99.4)
≥16	36.5 (27.3 – 46.6)	96.4 (93.4 – 98.2)	≥14	20.0 (12.8 – 28.9)	99.3 (97.4 – 99.0)
			≥15	14.3 (8.2 – 22.5)	99.6 (98.0 – 100)
			≥16	6.7 (2.7 – 13.3)	99.6 (98.0 – 100)

A potential source of criticism for the current analysis refers to the time after birth in which the screening was conducted. However, there is an incomplete knowledge regarding the natural history of PPD: while for the Diagnostic and Statistical Manual of Mental Disorders, 4^th ^ed. (DSM-IV) PPD has its onset within four weeks post-partum, others define onset up to the three months post-partum [13,14]. Therefore, the ideal timeframe for screening has not yet been established [15]. Most PPD research has used the second and third post-partum months for screening [5]. Furthermore, most instruments used to screen PPD were tested and showed to be valid and reliable when applied in the second and third post-partum months [16].

A recent meta-analysis reviewing articles printed in English and published up to December 2004, identified eight self-report questionnaires currently used for assessing depressive symptoms within the first year postpartum [13]. The EPDS, but not the SRQ20, was among them. The present study showed that SRQ20 and EPDS are very similar in performance to screen for PPD when assessed against the psychiatric interview taken as the gold standard. A previous study had already described a good agreement rate between EPDS and SRQ20, with a Kappa statistics of 0.75 [17]. Such findings support the validity of SRQ20 as a screening tool for PPD.

It should be noted that the highest sensitivity and specificity for SRQ20 as a screening tool for Brazilian women in general population was obtained with a cutoff of ≥ 8 [18]. For PPD, the corresponding SRQ20 cutoff point was lower, ≥ 6.

Characteristics regarding area of application and type of administration are very similar for EPDS and SRQ20. Both were developed for use in clinical and research settings and have the advantage of being suitable for use by lay interviewers in a face-to-face interview. Although EPDS has a smaller number of items than the SRQ20, the last can typically be applied by trained interviewers in a ten minute time [8].

The results of this study may be relevant for primary health care services and for future research. As PPD gains growing recognition as a public health concern and evidence of benefit of early treatment accumulates, the number of studies to identify the group of mothers in higher risk of PPD will increase. Due to its large use and the evidence of its adequate accuracy, the use of the SRQ20 as a screening tool for detecting PPD should also be considered.

## Competing interests

The author(s) declare that they have no competing interests.

## Authors' contributions

ISS conceived of the study, and participated in its design and drafted the manuscript. AM participated in its design and performed the statistical analyses. BFT participated in the design and coordination of the study and helped to draft the manuscript. ACCL participated in the design of the study and helped to draft the manuscript. RER and BCL participated in the coordination of the study. All authors read and approved the final manuscript.
